# A computational account of how individuals resolve the dilemma of dirty money

**DOI:** 10.1038/s41598-022-22226-9

**Published:** 2022-11-03

**Authors:** Jenifer Z. Siegel, Elisa van der Plas, Felix Heise, John A. Clithero, M. J. Crockett

**Affiliations:** 1 Department of Experimental Psychology, University of Oxford, Oxford, UK; 2 Department of Psychology, Yale University, 2 Hillhouse Ave, New Haven, CT 06511 USA; 3 Institude of Neurology, University College London, London, UK; 4 Lundquist College of Business, University of Oregon, Eugene, USA; 5 Department of Psychology and University Center for Human Values, Princeton University, Princeton, USA

**Keywords:** Human behaviour, Psychology

## Abstract

Money can be tainted when it is associated with direct or indirect harm to others. Deciding whether to accept “dirty money” poses a dilemma because money can be used to help others, but accepting dirty money has moral costs. How people resolve the dilemma of dirty money remains unknown. One theory casts the dilemma as a valuation conflict that can be resolved by integrating the costs and benefits of accepting dirty money. Here, we use behavioral experiments and computational modeling to test the valuation conflict account and unveil the cognitive computations employed when deciding whether to accept or reject morally tainted cash. In Study 1, British participants decided whether to accept “dirty” money obtained by inflicting electric shocks on another person (versus “clean” money obtained by shocking oneself). Computational models showed that the source of the money (dirty versus clean) impacted decisions by shifting the relative valuation of the money’s positive and negative attributes, rather than imposing a uniform bias on decision-making. Studies 2 and 3 replicate this finding and show that participants were more willing to accept dirty money when the money was directed towards a good cause, and observers judged such decisions to be more praiseworthy than accepting dirty money for one’s own profit. Our findings suggest that dirty money can be psychologically “laundered” through charitable activities and have implications for understanding and preventing the social norms that can justify corrupt behavior.

## Introduction

In early 2019, dozens of organizations began refusing charitable donations from the Sackler family, whose members stood accused of fueling the deadly opioid crisis. Later that year, a scandal erupted upon revelation that researchers at MIT and Harvard had accepted nearly a million dollars from convicted sex offender Jeffrey Epstein. These are just new examples of an old dilemma. The concept of “dirty money” dates back thousands of years, and can be defined as money associated with direct or indirect harm to others. Deciding whether to accept dirty money poses a dilemma because money can be used to help others, but accepting dirty money has moral costs^1^. How do people resolve this dilemma?

Research on “moral contagion” demonstrates that the value of objects can be tainted by association with moral misdeeds^2–4^. For example, participants will pay less money for items previously owned by immoral individuals^5,6^. There is also evidence that money itself is less subjectively valuable when it is obtained immorally. People imagine they would rather not receive money that is morally tainted^7^, and when faced with actual decisions to accept money from an experimenter in exchange for the experimenter inflicting painful electric shocks on oneself or another person, most people would rather receive money from an experimenter harming themselves over someone else^8^. Neuroimaging studies demonstrate that morally tainted money and objects are associated with lower activity in the brain’s valuation network^5,9,10^. However, it remains unknown what cognitive computations are employed during decisions about whether to accept or reject morally tainted cash.

A recently proposed theory casts the dilemma of dirty money as a *valuation conflict*^1^. According to this theory, when people are deciding whether to accept dirty money, they must resolve a conflict between its positive attributes (e.g., its ability to purchase desirable goods or help others) and negative attributes (e.g., harmful outcomes associated with the money). Strengthening positive attributes, or weakening negative attributes, can shift the balance towards accepting dirty money. The idea that people might shift the subjective value of a decision’s positive and negative attributes to resolve moral conflicts is not new. Past research has shown that people can maintain a positive self-image of themselves despite harmful actions by manipulating the value of the moral costs and personal benefits of the choice, allowing the individual to benefit from their immoral actions while maintaining a positive self-view^11^. Moral judgments are hypothesized to play a central role in assigning positive or negative value to attributes and subsequent decisions. For example, judging the *source* of the money to be morally bad is predicted to decrease the likelihood of accepting the money, while judging the *destination* of the money to be morally good is predicted to increase the likelihood of accepting the money^12–14^.

**Figure 1:**
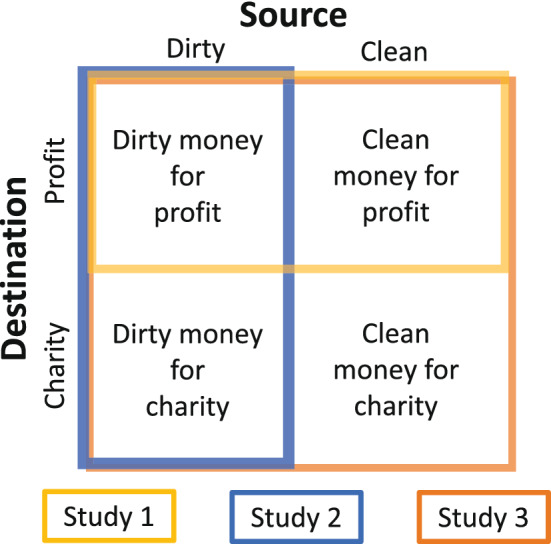
Overview of experimental design. Manipulation of source and destination as a function of study number. In Study 1 participants made moral decisions that varied as a function of the source (dirty vs. clean). In Study 2 participants made blame judgments about moral decisions that varied as a function of destination (profit vs. charity). In Study 3 participants made moral decisions that varied as a function of the source and destination.

**Figure 2:**
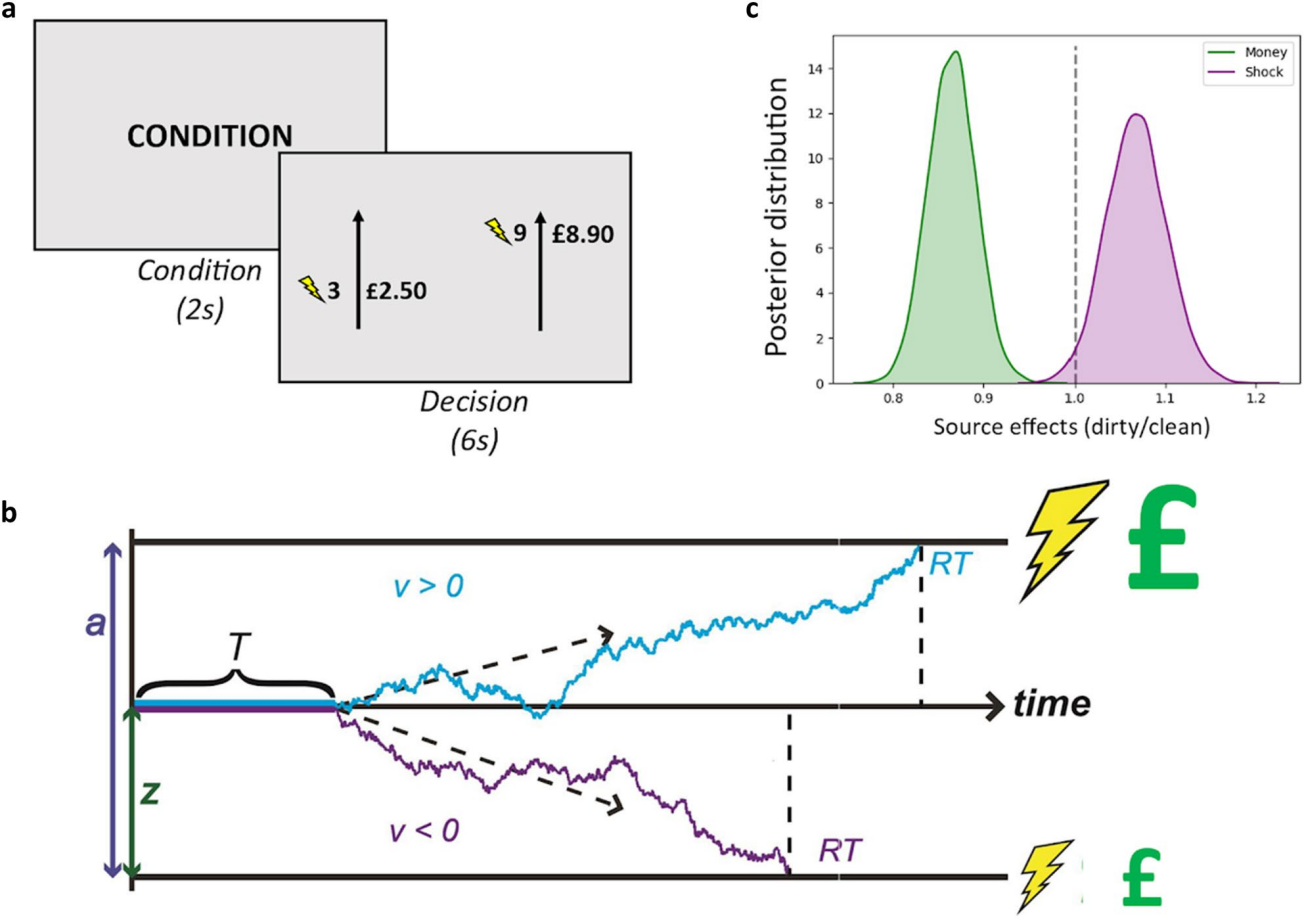
Moral decision-making task and modeling framework. (**a**) To probe moral decisions, participants (known as the “Decider”) made a series of real decisions, where each decision involved choosing between two options: a smaller amount of money plus a smaller number of painful electric shocks, or a larger amount of money plus a larger number of shocks. Before observing the choice options, a screen was presented indicating the recipient of the money and the shocks on the current trial. For half of the trials the shocks were allocated to the Decider and for the other half the shocks were allocated to an anonymous stranger in the next room. In study 1, the money recipient was always the Decider. In study 3, for half of the trials the recipient of money was the Decider and for half the trials the money was donated to a charity (Children with Cancer, UK). (**b**) Visual schematic for how the DDM captures choices and response times in Study 1 and Study 3. Responses are coded as larger shocks on the upper threshold and smaller shocks on lower threshold. Model parameters are discussed in the main text. (**c**) Moral decisions involving clean and dirty money (Study 1). The valuation of pain and money are sensitive to source effects: The weight on shocks was significantly higher when considering dirty relative to clean money (purple), while the weight on money was significantly lower when considering dirty relative to clean money (green).

Here, we tested the predictions of the valuation conflict hypothesis across a series of studies (summarized in Fig. 1). Across all studies we operationalized “dirty money” as money obtained by an experimenter inflicting painful electric shocks on another person, and “clean money” as money obtained by an experimenter inflicting shocks on oneself (Fig. 2a). In Study 1, participants decided whether to accept or forego different amounts of money which varied in their source (dirty or clean). In Study 2, we tested whether moral judgments of decisions to accept dirty money were sensitive to the destination of the money (personal profit or charitable donation). Finally, in Study 3, participants decided whether to accept or forego different amounts of money which varied in both their source and destination. The valuation conflict account predicts that people would have a lower probability of accepting dirty than clean money, and additionally, that directing dirty money towards a charitable cause will mitigate source effects, effectively “laundering” its value through the charitable act.

To decompose the cognitive processes that unfold during decisions about dirty money, in Studies 1 and 3 we modeled participants’ decisions and response time distributions using a multi-attribute extension of the drift–diffusion model (DDM)^15,16^. In this model, decisions to accept or reject money are compared by computing a subjective value over multiple attributes. Over time, this value integration represents accumulated value in favor of one option over another. A choice is made when the decision variable passes a threshold for one of the choice options.

By parameterizing several distinct aspects of the decision process, the DDM can also test the valuation conflict account of the dirty money dilemma (Fig. 2b). The drift rate parameter *v* captures the rate of value accumulation toward one of two options. This parameter relates to the efficiency at which the choice attributes (i.e., shocks and money) are processed. The bias parameter *z* captures the extent to which people lean toward accepting the larger amount of money before they know how much money is at stake. Therefore, the bias parameter *z* captures cognitive processing that occurs before the specific attributes of the choice are known. The cognitive difference between these two parameters is the extent to which the choice attributes affect the evaluation of the options (as is the case for the drift rate, v) or whether this evaluation is affected prior to observation of the attributes of the choice (as is the case for starting point bias, z). The parameter *a* represents the distance between the two decision thresholds, known as the decision boundary, *a*. If a decision maker is biased towards a particular response then *z* will not be equidistant between 0 and *a*. The non-decision time parameter, *T*, accounts for basic perceptual processing, such as recognizing that a choice has been presented, and captures any time required to initiate a response, such as pressing a button. The valuation conflict hypothesis^1^ focuses on the valuation process, and therefore predicts that source and destination effects should impact the drift rate parameter *v*. Here, we tested if the source of the money impacts the rate at which the valuation of money is accumulated, the rate at which the valuation of harm is accumulated, or both.

As the valuation conflict framework focuses on the valuation process, that is, the processing of the choice attributes (i.e., money and harm), it does not make predictions about the bias parameter, *z*. However, it is possible that people have a *prosocial default*^17,18^*,* whereby people are generally predisposed against increasing harm to others. This bias is independent of the specific attributes of the choice. The prosocial default account predicts that source and destination effects should be captured via the bias parameter *z,* biasing individuals towards foregoing dirty money even before they know the amount at stake. While theoretical accounts make a broad claim that behavior is prosocial as a “default” or “heuristic,” they have not specified how this is implemented at a computational level in terms of how choices are made. Here, we test whether in a two-alternative forced choice situation people are predisposed to choose the prosocial option before they receive any information about the precise attributes of that choice. Thus, using DDM we can arbitrate between temporally distinct latent processes in complex decision making; while source effects on starting point, *z*, suggest a predisposition to select one option over the other before observing the attributes of the choice, source effects on the drift rate, *v,* reflect valuation-based processes related to the evaluation of the choice attributes themselves. Consequently, using formal model comparison, we generate further insight into the predictions of the valuation conflict and prosocial default accounts by investigating the effects of source and destination on model parameters.

## Study 1

### Methods

First, we exploited a previously published dataset^9^ in which participants decided whether to accept “clean” money (obtained by inflicting painful electric shocks on themselves) or “dirty” money (obtained by inflicting painful electric shocks on another person) for their own personal profit. In each condition (dirty, clean), participants faced a series of decisions which varied in terms of how many shocks were imposed and how much money was obtained (Fig. 2a). We previously reported source effects on decision making in this dataset: participants in this experiment preferred taking clean money to dirty money^9^. Additionally, participants were slower to accept dirty relative to clean money. These results are consistent with both accounts of the dirty money dilemma: valuation conflict and prosocial default. In Study 1, we tested the predictions of each model by fitting multi-attribute DDMs using participants’ choices and response times (RT), as detailed below.

#### Participants

For Study 1, we conducted further analysis of a previously published dataset^9^ that included 28 participants aged 18–35 (12 female, mean age: 21.90 years), recruited from the University College London (UCL) Psychology Department and the Institute of Cognitive Neuroscience subject pools. All methods were carried out in accordance with relevant guidelines and regulations. Exclusion criteria included a history of systemic or neurological disorders, psychiatric disorders, psychoactive medication or drug use, pregnancy, more than two years' study of psychology, and previous participation in studies involving social interactions and/or electric shocks. Because this was an fMRI study, we recruited right-handed participants only. Because this was a previously published dataset, no statistical methods were used to pre-determine sample sizes. The sample size for the original study was based on estimated effect size for moral preferences observed in two previous behavioral studies using the same task^8^.

#### Moral decision task: source effects

Stimuli were presented using the Cogent toolbox (www.vislab.ucl.ac.uk/cogent.php) in MATLAB (MathWorks Inc.). We developed a moral decision-making paradigm^8,9,19^ where participants made 204 decisions that involved a trade-off between real monetary rewards and mildly painful electric shocks (Fig. 2a). In Study 1, across trials we manipulated *how* participants obtained money for profit (*source effects*): on half the trials the money was “clean,” obtained by the experimenter inflicting painful electric shocks on oneself. On the other half of trials, the money was “dirty,” obtained by the experimenter inflicting painful electric shocks on another. Before observing the choice options, a screen was presented indicating the recipient of the shocks on the current trial. This enabled us to test for any a priori bias individuals have towards foregoing dirty money even before they know the amount of money and shocks at stake.

Participants were instructed to indicate their preference within 6 s by pressing a button box with the left or right index finger. Any trial where the participant did not respond within 6 s was recycled and presented at the end of the task. The exact timing of decisions, the corresponding choice and the difference between the shock- and money-offers were used to simulate the valuation-process with the Python-based Hierarchical Drift–Diffusion Model toolbox (HDDM^20^), which will be discussed below.

Participants were instructed that at the end of the experiment one of their decisions would be selected at random and implemented.

#### Generation of choice options for the moral decision task

A single trial consisted of two options, each including a number of shocks (0 to 20) and an amount of money (£0 to £20). One option *l* was always associated with a lower number of shocks *s*_*l*_ and a lower amount of money *m*_*l*_ and the other option *h* with a higher number of shocks *s*_*h*_ and a higher amount of money *m*_*h*_. Initially, a set of 102 trials were created with each trial fitted to the indifference point of a simulated agent with a specific harm aversion coefficient κ. Values of κ were chosen equidistant from a uniform distribution covering the whole range of possible κ values (from 0 to 1). For each value of κ 1,000 random pairs of shock movement ∆s (i.e., the difference between the higher number of shocks *s*_*h*_ and the lower number of shocks *s*_*l*_) and money movement ∆m were created. The value of ∆s was restricted to positive integers ranging from 1 to 20 shocks and for ∆m ranging from 0.1 to 19.9 British pound sterling. The pair that was closest to the indifference point of the respective value of κ was then selected and transformed into a trial. This was done by giving the lower option a random *s*_*l*_ constrained by 0 ≤ *s*_*l*_ + ∆s ≤ 20, and a random *m*_*l*_ constrained by 0 ≤ *m*_*l*_ + ∆m ≤ 20. The higher options values were then simply given by *s*_*h*_ = *s*_*l*_ + ∆s and *m*_*h*_ = *m*_*l*_ + ∆m. To avoid confounding the effects of the specific trial set with the effect of the different conditions, the same set of trials was used in both conditions (dirty and clean). However, within each condition the trials were counterbalanced with regards to higher and lower options appearing on the left and right side of the screen. This was intended to make it more difficult for participants to notice repeating trials and to keep the task more interesting.

#### Computational framework for valuation conflict

Consistent with a growing body of work on value-based choices we assumed that choices can be captured using a multi-attribute variant of a drift–diffusion model (DDM)^15,16,21^, in which noisy value signals accumulate over time and a choice is made when the accumulated signal crosses a predefined threshold for choice. In this task, we assume that the relevant attributes of each option are the relative money (Δm) and relative shocks (Δs). The comparison process stops when a boundary (a), representing the relative evidence in favor of one of the options, is reached and the corresponding option is selected. We coded the decision upper and lower boundaries as corresponding to those for choices associated with the higher and lower amount of harm, respectively. According to a *valuation conflict framework,* the average drift rate (*v*) captures the overall speed of the accumulation process and varies from trial to trial with Δm and Δs, according to *drift weights* m and s, respectively:

$$v={\beta }_{m} \Delta m+{\beta }_{s}\Delta s$$

The accumulation of evidence starts from a starting point (z), which represents an a priori preference towards one of the choice options before any trial information is presented (e.g., a *bias* towards choosing the option with the smaller shock, regardless of shock magnitude). Models testing for a *prosocial default* suggests that source effects should impact the bias parameter *z,* biasing individuals towards foregoing dirty money even before they know the amount at stake. The DDM assumes RT is the sum of the duration of the decision time and a non-decision time component (T). Each of these parameters is depicted in Fig. 2b. Additional details on various DDM specifications are provided in the Supplementary Methods.

All DDM estimation was performed using a software package freely available in Python^20^. Models were estimated using a Bayesian hierarchical framework, with Markov chain Monte Carlo (MCMC) sampling methods employed to estimate a joint posterior distribution of the model parameters. Hierarchical Bayesian estimation allows individual participant estimates to be constrained by a group distribution, but also to vary to the extent that their data demonstrate separation from the data of others. Estimation used non-informative priors: all priors were uniform distributions over large intervals of possible parameter values. As outlier RT can cause estimation issues for the DDM^22^ we removed all trials with RT faster than 200 ms prior to model fitting.

We used two measures for model comparison. First, the Deviance Information Criterion (DIC) is a flexible measure for goodness-of-fit in hierarchical Bayesian models^23^. The DIC combines a measure of deviance (i.e., lack of fit) with a penalty for model complexity, and a lower DIC represents a better model fit. Second, we compared observed data to simulated data using the mean of sampled posteriors. For each model, 10,000 simulations were run with choice and RT data. The squared error between the observed data and the simulated data for each condition (e.g., shocks for self, shocks for other) was computed for both choices and RT, with the mean squared error (MSE) computed across conditions. This allowed us to identify how well a fitted model can recreate the observed data^24^. These statistics are summarized in Supplementary Results.

Model convergence was assessed using the Gelman-Rubin (G-R) R statistic^25^. The R statistic compares within-chain and between-chain variance of different runs of the same model. Perfect convergence across runs would result in an R of 1. The R statistic was computed for all of the model parameters based on five runs of the model^15^. These statistics are summarized in Supplementary Results.

Bayesian estimation also makes it straightforward to compare differences in parameter estimates. Significance inference is possible using the posterior distributions generated from the MCMC sampling process. To compare coefficients to the null hypothesis (β = 0) we assessed the percentage of the N = 10,000 sampled coefficients that were greater than zero. Similarly, to determine significant differences between two regression coefficients, we computed what percentage of samples indicated a difference that was greater than zero. In our analysis, we are particularly interested in the ratios of various parameters (e.g., Δm and Δs in various conditions). For these comparisons, we compared the ratios using the full posterior distribution of each ratio of interest.

### Results

To test how source affected decisions to accept money, we computed the proportion of decisions in each condition where participants chose to accept more money (at the expense of more shocks) and entered these proportions into a repeated-measures analysis of variance (ANOVA) with source (dirty vs. clean) as within-subject factors. We found that participants preferred accepting money from a clean rather than a dirty source when the money was for personal profit (*t*_27_ = 2.190, *p* = 0.037).

To test the predictions of the valuation conflict and prosocial default accounts of the dirty money dilemma, we fit multi-attribute DDMs using participants’ choices and response times (RT). The valuation conflict hypothesis predicts that the source of the money will impact the relative weighting of its positive and negative attributes, such that positive attributes (i.e., money) would outweigh negative attributes (i.e., harm) of decisions to accept clean money, while the reverse will be observed for dirty money. Meanwhile, the prosocial default hypothesis predicts that the source of the money will impact the starting point of the valuation process, with a starting point biased more towards ‘reject’ for dirty relative to clean money.

We formalized these hypotheses with separate models capturing different effects of source on model parameters. The prosocial default model fit separate starting point parameters for dirty and clean money. The family of valuation conflict models fit separate drift weights for positive and negative choice attributes for dirty versus clean money in various combinations (see Methods for details). As a baseline, we also fit a naïve model for which parameters did not vary at all by condition or trial.

We performed formal model comparisons along three dimensions, as outlined above. First, we used the DIC, a common measure for goodness-of-fit in hierarchical Bayesian models^23^. We also ran simulations using the fitted parameters to determine the extent the models could recreate the observed data^24^, looking at MSE between true and simulated data for both choice and RT. These three measures allowed us to triangulate the best fitting model. Additional model comparison details are provided in the Supplementary Materials.

Across all three measures of model fit, the prosocial default model performed worse than any of the valuation conflict models (DIC = 20,635.44, Choice MSE = 0.2163, RT MSE = 0.7695). The best-fitting model of those we tested was in the family of valuation conflict models, where drift rate is modeled as a multi-attribute combination of money and shocks, with distinctive weights on these attributes for dirty vs. clean money (DIC = 16,653.22, Choice MSE = 0.0992, RT MSE = 0.6788). As an additional test of the prosocial default hypothesis, we also modified the best-performing valuation conflict model to include separate starting points for dirty vs. clean money. This hybrid valuation-default model performed worse than the best-fitting valuation conflict model on all three dimensions (DIC = 16,662.66, Choice MSE = 0.0993, RT MSE = 0.6790), and the two starting point parameters were not significantly different from one another (*p* = 0.228). Overall, the model comparisons provide decisive support for the valuation conflict hypothesis and no evidence for the prosocial default hypothesis in this dataset.

We next tested a series of theoretical predictions by examining the parameter estimates from our best-fitting valuation conflict model. The valuation conflict hypothesis posits that decisions about dirty money involve the concurrent activation of conflicting impulses toward materiality and morality^1^. In the context of our model, this predicts that the source of money (dirty vs. clean) will impact the relative weights on positive and negative choice attributes (money and shocks, respectively). To test this, we compared the posterior distributions for the weights on money and shocks across source conditions (dirty relative to clean). Ratios significantly different from 1 indicate the presence of source effects on each attribute. Here, ratios significantly lower than 1 indicate that the drift weight for that attribute was lower when considering dirty relative to clean money, while ratios significantly greater than 1 indicate that the drift weight for that attribute was significantly higher when considering dirty relative to clean money. We found that the weights on money and shocks were *both* sensitive to source effects (Fig. 2c). The weight on shocks was significantly higher when considering dirty relative to clean money (mean ratio = 1.068, *p* = 0.0181), while the weight on money was significantly lower when considering dirty relative to clean money (mean ratio = 0.865, *p* < 0.001). To provide another test for the key ratio comparisons, we computed the 95% Highest Density Interval^26^, to determine if it overlapped with 1 or not. The HDI of the dirty/clean ratio for money did not overlap with 1 [0.8149,0.9185], nor did it for shocks [1.0014,1.1317]. This is consistent with the posterior distributions displayed in Fig. 2c.

Together, the findings from Study 1 reveal that source effects on decisions to accept money can be explained through a shift in the relative valuation of positive and negative choice attributes, consistent with the valuation conflict hypothesis. This suggests that resolving the dilemma of dirty money involves integrating over money’s positive and negative attributes, rather than adopting a heuristic strategy whereby deciders have an a priori bias against increasing harm to others and thus are inclined to reject any morally tainted money regardless of its attributes.

## Study 2

### Methods

Having found initial support for the valuation conflict hypothesis, we next turned to the possibility that altering the *destination* of dirty money could “launder” its value^27^. For example, donating dirty money to charity (rather than keeping it for oneself) could make initial decisions to accept dirty money more palatable, perhaps because such decisions might be seen as more morally justified. A prerequisite for testing this hypothesis is establishing that disinterested observers do in fact judge it to be less blameworthy to accept dirty money on behalf of a charity than to keep it for oneself. We tested our pre-registered prediction (https://aspredicted.org/blind.php?x=zb65vc) that participants would judge decisions to take dirty money as more blameworthy when the destination of the money was personal profit relative to charitable donation using a Moral Judgment Task.

#### Participants

For Study 2, 152 UK university students between the ages of 18 and 35 were recruited from the online crowdsourcing platform, Prolific (www.prolific.ac), and randomized to either a profit condition or a charity condition. All participants provided written informed consent prior to participation and were compensated for their time. The Yale University Human Investigation Committee approved the procedures, ethics number 2000022385. All methods were carried out in accordance with relevant guidelines and regulations. Eleven participants randomized to the profit condition and eleven participants randomized to the charity condition were excluded from the analysis for failing pre-registered attention checks (see below). Final analysis was carried out on the remaining 64 participants in the profit condition (40 female, mean age: 22.48 years) and 66 participants in the charity condition (49 female, mean age: 22.74 years). An a priori power analysis indicated that the study required 64 participants in each condition to have 80 percent power to detect a moderate effect (0.5) in a parametric between-groups analysis. Thus, our study was sufficiently powered to observe an effect in our between-groups design.

#### Moral judgment task

The moral judgment task implemented in Study 2 asked participants to judge how blameworthy they thought it would be for an agent (known as a ‘Decider’) to make each of 42 moral decisions. For each decision, the Decider chose between a smaller amount of money plus a smaller number of electric shocks for a stranger (helpful option), or a larger amount of money plus a larger number of electric shocks for the stranger (harmful option), as shown in Fig. 2a. We parametrically modulated the amount of money and shocks in each trial to produce a series of choices that varied in their relative harmfulness (see *Generation of choice options for the Moral Judgment Task* below). Upon seeing each pair of options, participants judged how blameworthy they thought it would be if a Decider chose the more harmful option (i.e., the larger amount of money and shocks) on a continuous scale ranging from 0 (*extremely praiseworthy*) to 100 (*extremely blameworthy*).

#### Generation of choice options for the moral judgment task

A single trial consisted of two options, each including a number of shocks (1 to 20) and an amount of money (£0.10 to £20). One option *l* was always associated with a lower number of shocks *s*_*l*_ and a lower amount of money *m*_*l*_ and the other option *h* with a higher number of shocks *s*_*h*_ and a higher amount of money *m*_*h*_. A set of 42 trials were created with each trial fitted to the indifference point of a simulated agent with a specific price value for shocks. This value was parameterized as a ‘harm aversion’ coefficient κ. Values of κ were chosen equidistant from a uniform distribution covering the whole range of possible κ values (0 to 1). For each value of κ 1000 random pairs of shock movement ∆s (i.e. the difference between the higher number of shocks *s*_*h*_ and the lower number of shocks *s*_*l*_) and money movement ∆m were created. The value of ∆s was restricted to positive integers ranging from 1 to 20 shocks and for ∆m ranging from 0.1 to 19.9 British pound sterling. The pair that was closest to the indifference point of the respective value of κ was then selected and transformed into a trial. This was done by giving the lower option a random *s*_*l*_ constrained by 1 ≤ *s*_*l*_ + ∆s ≤ 20, and a random *m*_*l*_ constrained by 0.1 ≤ *m*_*l*_ + ∆m ≤ 20. The higher options values were then simply given by *s*_*h*_ = *s*_*l*_ + ∆s and *m*_*h*_ = *m*_*l*_ + ∆m. We ensured that ∆m and ∆s did not covary across trials (Pearson’s R = 0.004, *p* = 0.978).

#### Attention check

We included two attention check questions mixed within the 42 moral judgments. For each attention check, we presented participants with a pair of options, and instead of asking participants to judge how blameworthy they think it would be if a Decider chose the more harmful option, we asked participants to respond on the rightmost side of the scale (100 = *extremely blameworthy*). Participants who did not respond on the rightmost side of the scale for either of these questions were excluded from the analysis.

#### Manipulation

To investigate whether moral judgments were sensitive to the destination of the dirty money participants were randomly assigned to one of two conditions: a profit condition, where for each decision the Decider receives the money, or a charity condition, where for each decision the money is donated to Children with Cancer UK, a leading charity organization that helps provide better care for children with cancer and their families, and supports research into causes, prevention and treatment of childhood cancer. Accordingly, participants in the profit condition judged harmful decisions that benefit the Decider personally, while participants in the charity condition judged harmful decisions that benefit a charity.

#### Analysis

All data analysis was completed in Matlab (Mathworks) and all statistical tests were two-sided. We hypothesize that participants in the profit condition will assign greater blame for harmful decisions than participants in the charity condition. To test our primary preregistered hypothesis that people assign greater blame for ill-gotten harms than harms for the greater good, we carried out a between-subjects parametric test (independent samples *t*-test) on subjects’ average blame rating across trials. As secondary preregistered analyses, we used a linear mixed effects model with a random intercept to model how money and shocks in each choice option predict blame judgments, and whether the effects of money and shocks on blame judgments differ as a function of the money recipient. The regressors in the model included: the difference between the chosen and unchosen amount of money (Δ*m, β*_*1*_), the difference between the chosen and unchosen amount of shocks (Δ*s, β*_*2*_), the money recipient (‘condition’, dummy coding for profit versus charity, *β*_*3*_), the interaction between condition and Δ*m* (*β*_*4*_), and the interaction between condition and Δ*s* (*β*_*5*_), and a random intercept for participants (*u*_*0i*_). Our regression included a fixed intercept term (*β*_*0*_), capturing the average judgment across trials, and all other coefficients expressed mean deviations from this judgment. Parameters were estimated using maximum likelihood estimation.

$$Blame={(\beta }_{0}+ {u}_{0i})+{\beta }_{1}\left(\Delta m\right)+{\beta }_{2}\left(\Delta s\right)+{\beta }_{3}\left(condition\right)+{\beta }_{4}\left(condition*\Delta m\right)+ {\beta }_{5}\left(condition*\Delta s\right)$$

We report means and standard error of the mean (sem) as mean ± sem. Effect sizes were computed for significant results using Cohen’s *d*.

### Results

**Figure 3:**
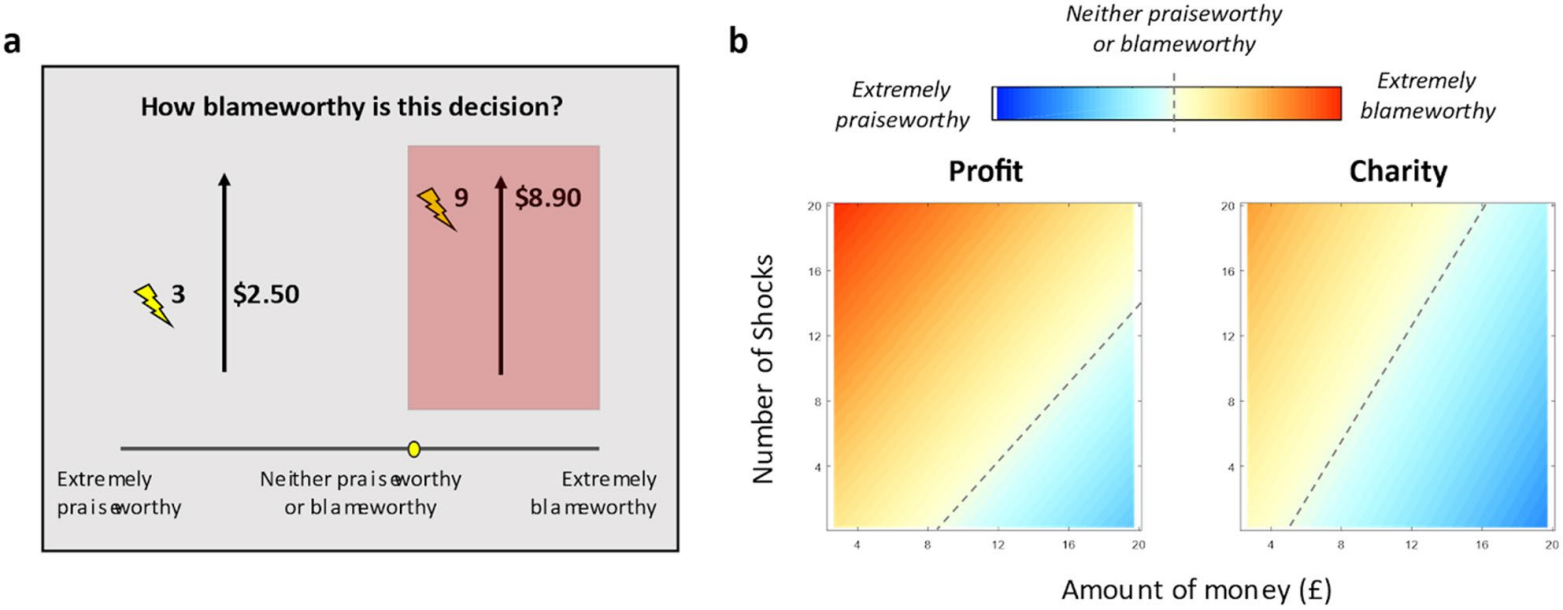
Moral judgment task and destination effects on blame. (**a**) In Study 2 participants rated how blameworthy they thought it would be if a person chose the more harmful option (i.e., the larger amount of money and shocks, highlighted in red) on a continuous scale ranging from 0 (extremely praiseworthy) to 100 (extremely blameworthy). (**b**) Estimated blame judgments from a linear mixed-effects model as a function of money and shocks. Blame for accepting dirty money is sensitive to the destination of the money. Dashed diagonal lines indicate mix of money and shocks that are neither praiseworthy nor blameworthy.

We fit a linear mixed-effects model to describe how blame judgments varied as a function of the amount of harm imposed, the amount of money obtained, and the destination of the money (see Methods for details and Supplementary Table 4 for results of the full model). Consistent with past work (Siegel, Crockett, & Dolan, 2017), across both conditions, blame judgments were positively correlated with the amount of harm (*β* = 1.697 ± 0.081, *t* = 20.894, *p* < 0.001) and negatively correlated with the amount of money (*β* = -1.879 ± 0.078, *t* = -24.211, *p* < 0.001), Fig. 3a. As we predicted, participants in the profit condition assigned significantly more blame overall than participants in the charity condition (mean ± sem, profit: 62.842 ± 0.215; charity: 49.543 ± 0.204; *t* = 5.563, *p* < 0.001, *d* = 0.976). To illustrate these effects, we fit the linear mixed-effects model separately for the profit and charity condition and plotted the estimated blame judgments as a function of money and shocks in Fig. 3b. The heatmap for Profit (left) demonstrates an increase in blame relative to the Charity (right) condition. To summarize, study 2 demonstrates that blame for accepting dirty money is sensitive to the destination of the money, where blame is mitigated when dirty money is directed toward a charitable cause.

## Study 3

Following the results of Study 1, which supported the valuation conflict account, we predicted that participants would prefer to accept clean over dirty money for their own personal profit, but that source effects would be reduced when the money was destined for a charity. Furthermore, we expected that both source and destination would impact the relative weighting of the positive and negative attributes of money. Specifically, we expected to replicate the source effects observed in Study 1, such that decisions about dirty relative to clean money would involve higher weights on shocks and lower weights on money. Following the results of Study 2, we expected that directing dirty money toward charity would reduce the impact of negative attributes on the valuation of money.

### Methods

Study 1 provided initial support for the valuation conflict hypothesis, demonstrating that decisions about whether to accept dirty (versus clean) money involve integrating across the money’s positive and negative attributes. Study 2 showed that accepting dirty money is less blameworthy when the money is directed to charity than when it is kept for personal gain. In Study 3, we build on these findings to further test the predictions of the valuation conflict and prosocial default accounts of the dirty money dilemma. The valuation conflict account, but not the prosocial default account, predicts that directing dirty money to charity can effectively “launder” its value. Consequently, we adapted the Moral Decision Task implemented in Study 1 to test the effects of both source (dirty, clean) and destination (profit, charity) on moral decision making.

#### Participants

For Study 3, healthy participants aged 18–35 were recruited from the University of Oxford’s Department of Psychology subject pool. Participants with a history of neurological or neuropsychiatric disorders, pregnant women, and more than one year of study in psychology were excluded from participation. Participants who had previously participated in studies involving deception of electric shocks were also excluded due to concerns that prior experience with being deceived would influence belief in the outcomes of the current task, which did not involve deception. All participants provided written informed consent and were compensated for their time. Study 3 was approved by the University of Oxford ethics committee (R50262/RE001). All methods were carried out in accordance with relevant guidelines and regulations.

Participants in Study 3 attended a single testing session at the University of Oxford’s Department of Experimental Psychology. Sixty-two pairs of participants took part in the study. The two participants arrived at staggered times and were led to separate testing rooms without seeing one another to maintain anonymity. After providing informed consent, a pain thresholding procedure was used to familiarize participants with the electric shock stimuli prior to the task. Next, participants were randomly assigned to either the role of the ‘Decider’ or the ‘Receiver’. Receiver participants completed a moral learning task (data to be reported separately) and decider participants completed a moral decision task, which we focus on here. One decider participant did not complete the task due to a technical error, and therefore was excluded from the analysis. This left a total of sixty-one participants in the role of the decider whose data were analyzed (25 female, mean age: 23.95 years). A power analysis indicated that the study required 34 participants in each condition to have 80 percent power to detect a moderate effect (0.5) in a parametric within-groups analysis of dirty versus clean money. Thus, our study was sufficiently powered to observe source effects when money is destined for profit and for charity.

#### Moral decision task: source and destination effects

Stimuli were presented using the Cogent toolbox (www.vislab.ucl.ac.uk/cogent.php) in MATLAB (MathWorks Inc.). In Study 3, participants made 176 decisions in the Moral Decision Task (Fig. 2a). As in Study 1, across trials we manipulated *how* participants obtained money for profit (*source effects*): on half the trials the money was “clean,” obtained by the experimenter inflicting painful electric shocks on oneself. On the other half of trials, the money was “dirty,” obtained by the experimenter inflicting painful electric shocks on another person. In this study, we additionally manipulated *who* obtained the money across trial (*destination* effects): on half of the trials the money was for the decider (‘profit’ condition) and on the other half of trials the money was donated to a charity organization for children with cancer (‘charity’ condition). This resulted in a 2 × 2 factorial design in Study 3, with source (dirty vs. clean) and destination (profit vs. charity) as the independent variables. Closely matching the instructions given in Experiment 1, before observing the choice options, a screen was presented indicating the recipient of the shocks (self, other) and money (profit, charity) on the current trial. This allowed us to test for a tendency towards harming for charity before participants knew what amount of money and shocks were at stake.

As in Study 1, participants were instructed that at the end of the task, one of their decisions would be selected at random and implemented at the end of the experiment. This allowed us to ensure that any effort exerted in the process of inflicting shocks did not affect the valuation. Information about the charity was derived from its website (www.childrenwithcancer.org.uk) and presented to the participants in the following form: “The decisions you make during [charity] trials affects how much money we donate to Children with Cancer UK. This organization is a leading charity in the UK that helps provide better care for children with cancer and their families, and supports research into causes, prevention and treatment of childhood cancer in the UK.”

Trials were generated using an identical procedure to those outlined in Study 1. However, in Study 3, the trials from each condition were randomly sorted into sections of 10 trials. One section from each of the four conditions (see Fig. 1) were combined to form a block of 40 trials. These blocks were then randomly presented one after another with no block starting with the same condition that the previous block ended with.

#### Computational framework for valuation conflict

In addition to allowing the drift weights *v* to vary as a function of source, Study 3 fit models that allowed *v* to vary as a function of destination. Additional details on various DDM specifications are provided in the Supplementary Methods.

### Results

**Figure 4:**
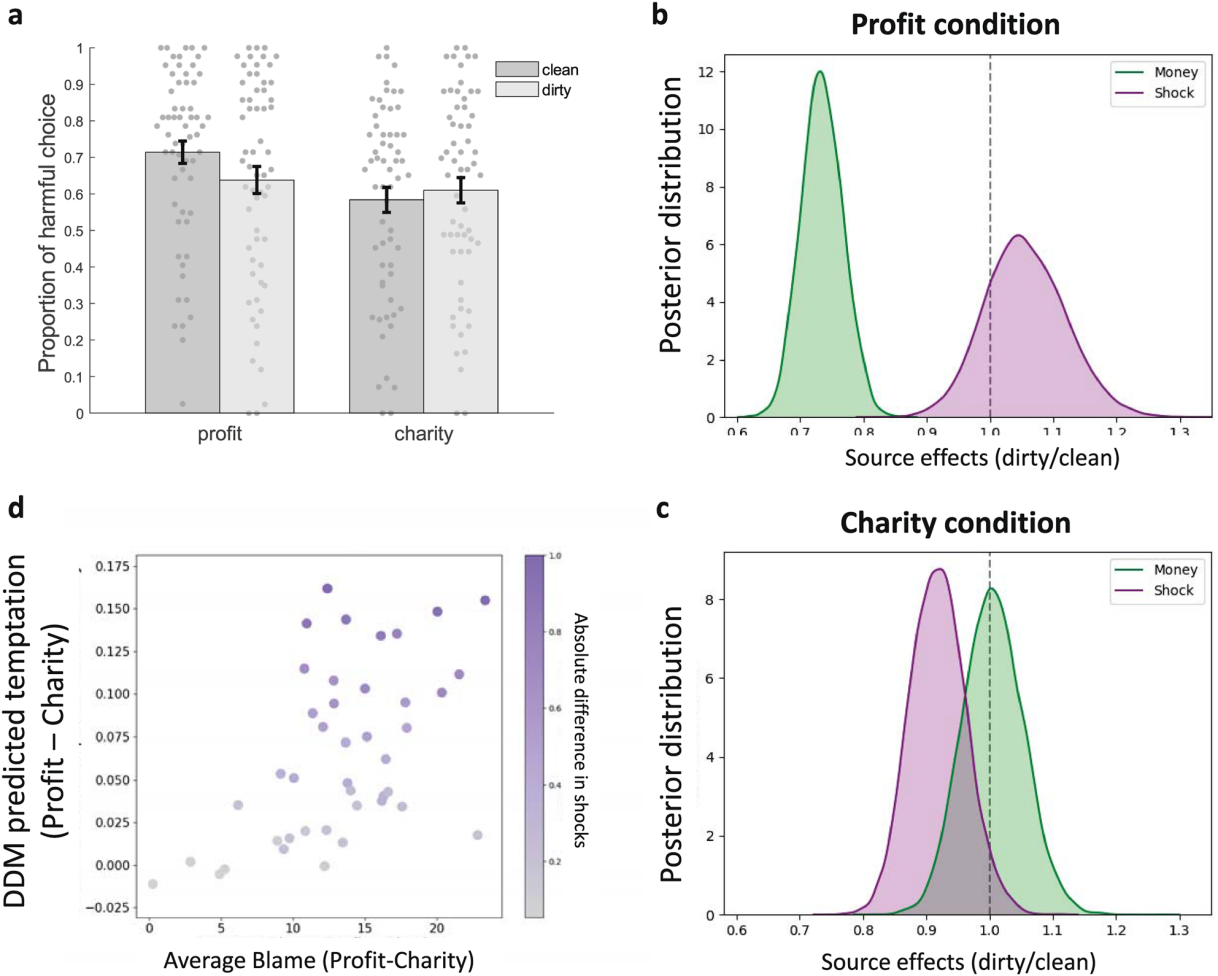
Source and destination effects on moral decision making. (**a**) Proportion of harmful choices made as a function of the source (dirty/clean) and destination (profit/charity) of the money. (**b**) In line with Study 1 (Fig. 2c) the weight on money was significantly lower when considering dirty relative to clean money for profit. (**c**) The weight on shocks was significantly lower when considering dirty relative to clean money for charity. (**d**) Scatterplot showing out-of-sample correlation between differences in blame judgments (Profit-Charity) in Study 2 (horizontal axis) and the estimated differences in value associated with choosing the more harmful option (Profit-Charity) in Study 3 (vertical axis). Each dot represents a possible combination of money and shocks offers. Dots are color coded according to the highest/lowest shock offers. Error bars represent standard error of the mean.

To test how source and destination affected decisions to accept money, we computed the proportion of decisions in each condition where participants chose to accept more money (at the expense of more shocks) and entered these proportions into a repeated-measures analysis of variance (ANOVA) with source (dirty vs. clean) and destination (profit vs. charity) as within-subject factors. We found that harm was mitigated when the money was destined for a charitable cause versus for profit (effect of destination: F_1, 60_ = 11.627, *p* = 0.001). Decisions to harm were not significantly affected by the source of the money (effect of source: F_1, 60_ = 0.0835, *p* = 0.365). However, as predicted, we observed a significant interaction between source and destination (interaction: F_1, 60_ = 30.929, *p* < 0.001). Simple effects analyses indicated that participants preferred accepting money from a clean rather than a dirty source when the money was for personal profit (*t*_60_ = 2.850, *p* = 0.006), but not when the money was for charity (*t*_60_ = − 0.851, *p* = 0.398, Fig. 4a. Furthermore, participants preferred accepting larger amounts of money/shocks for profit relative to for charity only when the money was clean (t_60_ = 4.8637, *p* < 0.001), but not when the money was dirty (t_60_ = 1.216, *p* < 0.229). This suggests that people may be particularly averse to their own harm when it benefits another, as opposed to themselves.

Again, we fit a series of DDM specifications to assess the cognitive computations employed when deciding whether to accept dirty money. The prosocial default model, as in Study 1, allowed the starting point parameter to vary across source conditions. The family of valuation conflict models fit separate drift weights for positive and negative choice attributes across source and destination conditions. Consistent with Study 1, we found evidence in support of the valuation conflict hypothesis. On all three model comparison indices, the valuation conflict model performed better than any other model (DIC = 35,535.33, Choice MSE = 0.2499, RT MSE = 0.6355). The best-fitting model was a valuation conflict model that allowed the drift rate to vary in terms of both source and destination effects (DIC = 28,023.20, Choice MSE = 0.1170, RT MSE = 0.5900). This model had separate parameters for drift weights on money and shocks in each of the four experimental conditions. As an additional test of the prosocial default account, we modified the best-fitting valuation conflict model to include separate starting points for source effects. As in Study 1, this hybrid valuation-default model performed worse on all three dimensions (DIC = 28,091.39, Choice MSE = 0.1171, RT MSE = 0.5910).

We next examined the parameter estimates from the best fitting model to test the effects of source and destination on the relative weighting of positive and negative attributes in decision making. We first compared parameter estimates for dirty versus clean money separately for each destination condition (Fig. 4b,c). In the profit condition, as in Study 1, the weight on money was lower for dirty relative to clean money (mean ratio = 0.735, *p* < 0.001 compared to ratio of 1). In contrast to Study 1 however, there was no evidence for source effects on the weighting of shocks (mean ratio = 1.056, *p* = 0.189). Moreover, the difference in the size of these effects was significant, such that source impacted the weighting of money to a significantly greater degree than the weighting of shocks (*p* = 0.011). Conversely, in the charity condition, no source effects were observed on money weights for dirty relative to clean (mean ratio = 1.006, *p* = 0.457). However, source impacted the weight on shocks, which was lower when accepting dirty, relative to clean, money for charity (mean ratio = 0.919, *p* = 0.036). Collectively, these findings demonstrate that considering whether to accept dirty money for profit is associated with a diminished valuation of money. However, when dirty money is directed toward a charitable cause (i.e., “laundered”), this reduction in value is no longer observed. Quantitatively, the impact of source on the weighting of money was approximately four times larger in the profit condition than the charity condition.

To provide another test for the key ratio comparisons, we computed the 95% HDI^26^, to determine if it overlapped with 1 or not. In the profit condition, the 95% HDI for the dirty/clean ratio of money did not overlap with 1 [0.6701, 0.7977], whereas for shocks it did [0.9358,1.1838]. This result is consistent with the above findings indicating a significant effect of source on money but not shocks in the profit condition. For the charity condition, the 95% HDI for money did overlap with 1 [0.9140,1.1012], which is consistent with our previous findings suggesting no effects of source on the weighting of money in the charity condition. However, we find a weaker result for shocks, as the 95% HDI slightly overlapped with 1 [0.8360, 1.0076].

Finally, as an out-of-sample check on the validity of the latent model parameters, we tested if there was a correlation between the blame judgments collected in Study 2 and the estimated value of accepting dirty money in Study 3. As noted in Study 2, participants judged it to be more blameworthy to accept dirty money for profit compared to charity. Here, we compared this gap in blame judgments between the profit and charity conditions to the “temptation” of selecting the option with more money in Study 3. “Temptation” was computed as the weighted sum of the difference in money and the difference in shocks, using the estimated DDM drift weights from the best-fitting model. To compute the gap in blame judgments between profit and charity conditions we computed the difference in the estimated blame for each possible combination of shocks and money, given the extracted beta weights from our linear mixed-effects models that were fit separately for the two conditions in Study 2.

$$Gap\, in \,blame \,judgement={Blame}_{profit}- {Blame}_{charity}$$

where:

$${Blame}_{profit}={\beta }_{1 profit}\left(\Delta m\right)+{\beta }_{2 profit}\left(\Delta s\right)$$

$${Blame}_{charity}={\beta }_{1 charity}\left(\Delta m\right)+{\beta }_{2 charity}\left(\Delta s\right)$$

Each possible combination of money and shocks is plotted in Fig. 4d. We observed a significant positive correlation between “temptation” and Profit-Charity blame (Pearson r = 0.520, *p* < 0.001). Furthermore, this positive correlation was tightly coupled to the underlying difference in shocks (Pearson r = 0.544, *p* < 0.001), as shown in the color coding of the dots in Fig. 4d. No such relationship existed with the amount of money (Pearson r = 0.100, *p* = 0.528, see corresponding plot in Supplementary Results). This demonstrates a proof of principle that the attribute weights assigned to available options predict both moral decisions and RT in the decision-making task, as well as moral judgments in the blame task^28^.

## Discussion

Folk intuitions and empirical evidence show that people are generally disinclined to accept dirty money, but sometimes do so anyway^7,8^. However, it remains unclear how people resolve the dilemma of dirty money. The valuation conflict hypothesis suggests people trade off the moral and material costs and benefits of dirty money^1^. By analyzing patterns of decisions and response times with multi-attribute drift–diffusion models, we find evidence to support this hypothesis. Participants in our experiments were more likely to accept dirty money when it was directed toward a charitable cause, suggesting that moral destinations can “launder” dirty money. Response time data showed that the source and destination of money impacted the accumulation of value during the decision process (as predicted by the valuation conflict account), but did not influence the starting point of value accumulation or the decision boundary. Together these findings suggest that individuals resolve the dilemma of dirty money by integrating over money’s positive and negative attributes.

In addition to providing empirical support for the valuation conflict framework, our findings further suggest that resolution of the conflict is accomplished primarily via changing the valuation of positive rather than negative attributes. Across Studies 1 & 3 we found consistent evidence that the source of money impacts the weight of positive attributes in the valuation process, but inconsistent evidence that source impacts the weight of negative attributes. These findings are consistent with past neuroimaging results^9^, which suggest that the decision to forego ill-gotten gains is driven by decreased activation in profit-sensitive brain regions (dorsal striatum) rather than increased activation in pain-sensitive brain regions (insula and anterior cingulate cortex). Together, the results enrich previous work in non-social decision making^9,29,30^ by further demonstrating that moral decision making shares the principles of information integration found in many other domains^16^.

Our results also reveal how dirty money can be “laundered” when it is directed towards a noble cause. Accepting dirty money that is meant for a charity is judged to be less blameworthy than using such money for personal gain (Study 2; Fig. 3b), and dirty money is devalued less when the money is for charity relative to when it is for profit (Study 3; Fig. 4b,c). These findings are consistent with previous studies showing that individuals will spend dirty money on more virtuous items as opposed to more hedonic ones^3^. Indeed, moral preferences are malleable to contextual changes that justify antisocial behavior: for example, people often donate less to charity if stinginess does not harm their reputation^31,32^. Our modeling work shows a plausible decision process that leads to such behaviors: changing the moral consequences of decisions alters the integration of distinct attributes^10^. Looking across studies, our findings provide evidence for the impact of third-party moral judgments on the formation of independently made moral decisions.

By studying moral judgment and moral decision making within the same experimental framework, we were able to explicitly link these cognitive processes^28^ to examine how people resolve the dilemma of dirty money. The valuation conflict account suggests that strengthening the positive attributes of dirty money can make people more likely to accept it. Here we show that making those positive attributes *moral* – for example, by directing the money towards a charity rather than one’s own profit – changes the way that money is valued. This process may operate via changing people’s beliefs about how third parties evaluate decisions to accept dirty money. Supporting this idea, we found that drift rates derived from our computational model of the decision-making process made accurate out-of-sample predictions regarding third parties’ blame judgments about decisions to accept dirty money (Fig. 4d). These findings suggest that people might simulate how observers would judge their decisions about whether to accept dirty money, and adjust their own decisions in tune with the estimated moral consequences of their decisions^33^. More broadly, this perspective suggests that integrative appraisals may be involved in understanding one’s own preferences (‘thinking about thinking’) and affect the subjective decision-making process accordingly^34^.

Our findings also align with a recent study by Qu and colleagues^14^, who asked participants to decide whether to accept money for themselves or a charity, at the expense of a moral cost (profiting a morally bad cause). They found that participants were more willing to accept ill-gotten gains for themselves than for a charity, which was driven by a diminished valuation of the negative attributes (i.e., moral costs) when deciding for their own interests. Similarly, Study 3 demonstrates that participants are also more willing to accept negative outcomes when deciding for their own interests, versus the interests of a charity. However, this was primarily associated with a diminished valuation of the decision’s positive attributes (i.e., money) when they came at a moral cost (i.e., shocks for another), as opposed to the decision’s negative attributes. Several differences across studies might explain this discrepancy, such as the stimuli used (shocks vs. profiting a morally bad cause) and the number of experimental manipulations (source and destination vs. destination alone). Future research is needed to elucidate the discrepancies between studies and determine under what conditions a willingness to accept ill-gotten gains for one’s own interest, over another’s, is driven by increased valuation of the decision’s positive attributes, versus diminished valuation of the decisions negative attributes.

One important limitation of our design is that our experimental context, trading money for electric shocks, is unlikely to be encountered in real life. Notably, while electric shocks are aversive, the stimulation was relatively mild and monetary rewards were small. Whether individuals resolve the dilemma of dirty money with similar cognitive computations in more familiar, real-world situations, e.g., stolen money or drug money, or when more extreme harms and money are at stake, is unknown. Past work has shown similar devaluation effects of dirty money in vignettes using other forms of dirty money, such as stolen money^7^, which suggests there might be a common mechanism in more familiar contexts. Additionally, our design requires participants to impose some amount of harm regardless of their decision. The cognitive computations underlying dirty money decisions may be different when deciding between inflicting some amount of harm for money versus no harm at all, i.e., zero shocks^35^. Future work should investigate whether an option where harm can be avoided entirely changes the decision dynamics as measured with the DDM.

Although our computational models fit latent parameters at the individual level, our hypothesis tests focus on the group-level effects of source and destination. Using these group-level parameters, we found that resolving the conflict of dirty money operates primarily via changing the valuation of positive rather than negative attributes. However, these group level findings may obscure systematic individual differences. For instance, some individuals may resolve the conflict via a response bias against increasing harm to others, consistent with the prosocial default account. Such individual differences may relate to other aspects of moral cognition, such as deontological versus utilitarian preferences. Those who rely on more rule-based strategies, such as those applied in deontology, may be more likely to resolve the conflict by adopting a prosocial default, while those who adhere to utilitarian preferences may be more likely to weigh up the positive and negative attributes of the conflict. Future work could use a combination of electroencephalogram (EEG) recordings and the DDM^36^, to obtain the temporal resolution required to infer if certain aspects of the moral choice are considered earlier in the valuation process.

Our paradigm taps into how people make decisions about money associated with harm to others. This reflects a shared aspect of many real-world cases of dirty money, for example, institutions that had to decide whether to accept money from the Sackler family associated with the misfortune of others during the opioid crisis. That said, there are many differences between our paradigm and real-world examples of “dirty money.” For instance, our paradigm may additionally tap into preferences for money that feels “earned” relative to money that feels “unearned.” Future research could tease apart these possibilities by comparing behavior in the present paradigm with one where participants make decisions about money they earn themselves (e.g., by exerting effort^37^), versus money earned through others’ effort.

Many transactions in our economy involve morally tainted goods. Our findings suggest multiple routes to target in the decision-making process with the ultimate goal of inducing behavioral change. For example, past work on value-based decision-making shows that attention and the valuation of choice attributes are tightly linked^21,35,36,38,39^. In the context of the current study, this suggests that drawing individuals’ attention to the harms of dirty money might reduce the likelihood of its acceptance. Similarly, we speculate that appraisal processes could play a role in guiding decisions involving dirty money. Narratives that highlight the potential positive impact dirty money can have on others (e.g., ill-gotten charitable contributions) may be especially enticing^40^. Therefore, developing interventions to shift individuals’ appraisals of dirty money from “good” to “bad” may increase rejections of dirty money.

In sum, we provide a computational characterization of cognitive processes that drive moral decisions involving dirty money. Our account of how these decisions unfold helps unpack an issue often discussed after a choice has been made. Understanding how dirty money can be psychologically “laundered” through charitable activities is an initial step towards the development of interventions that can help prevent the cycle of corrupt behavior.

## Supplementary Information

Supplementary Information.

## Data Availability

Studies 1 and 3 were not formally preregistered. All methods and analyses were preregistered for Study 2 and can be accessed at https://aspredicted.org/blind.php?x=zb65vc. All data, materials, and analysis code are available via OSF https://osf.io/qg8m7/.

## References

[1] Tasimi A, Gross JJ (2020). The dilemma of dirty money. Curr Dir Psychol Sci.

[2] Huang JY, Ackerman JM, Newman GE (2017). Catching (up with) magical contagion: A review of contagion effects in consumer contexts. J. Assoc. Consum. Res..

[3] Levav J, Mcgraw AP (2018). Emotional accounting: How feelings about money influence consumer choice. J. Mark. Res..

[4] Stellar JE, Willer R (2014). The corruption of value: Negative moral associations diminish the value of money. Soc. Psychol. Personal. Sci..

[5] Liu J, Liao C, Lu J, Luo Y, Cui F (2019). Moral contagion: Devaluation effect of immorality on hypothetical judgments of economic value. Hum. Brain Mapp..

[6] Newman GE, Bloom P (2014). Physical contact influences how much people pay at celebrity auctions. PNAS.

[7] Tasimi A, Gelman SA (2017). Dirty money: The role of moral history in economic judgments. Cognit. Sci..

[8] Crockett MJ, Kurth-Nelson Z, Siegel JZ, Dayan P, Dolan RJ (2014). Harm to others outweighs harm to self in moral decision making. PNAS.

[9] Crockett MJ, Siegel JZ, Kurth-Nelson Z, Dayan P, Dolan RJ (2017). Moral transgressions corrupt neural representations of value. Nat. Neurosci..

[10] Qu C, Météreau E, Butera L, Villeval MC, Dreher J-C (2019). Neurocomputational mechanisms at play when weighing concerns for extrinsic rewards, moral values, and social image. PLoS Biol..

[11] Mazar N, Amir O, Ariely D (2008). The dishonesty of honest people: A theory of self-concept maintenance. J. Mark. Res..

[12] Erat S, Gneezy U (2012). White lies. Manage. Sci..

[13] Yin L, Hu Y, Dynowski D, Li J, Weber B (2017). The good lies: Altruistic goals modulate processing of deception in the anterior insula. Hum. Brain Mapp..

[14] Qu C, Hu Y, Tang Z, Derrington E, Dreher J-C (2020). Neurocomputational mechanisms underlying immoral decisions benefiting self or others. Soc. Cognit. Affect. Neurosci..

[15] Harris A, Clithero JA, Hutcherson CA (2018). Accounting for taste: A multi-attribute neurocomputational model explains the neural dynamics of choices for self and others. J. Neurosci..

[16] Ratcliff R, Smith PL, Brown SD, McKoon G (2016). Diffusion decision model: Current issues and history. Trends Cogn. Sci..

[17] Rand DG (2016). Cooperation, fast and slow: Meta-analytic evidence for a theory of social heuristics and self-interested deliberation. Psychol. Sci..

[18] Zaki J, Mitchell JP (2013). Intuitive prosociality. Curr. Dir. Psychol. Sci..

[19] Crockett MJ (2015). Dissociable effects of serotonin and dopamine on the valuation of harm in moral decision making. Curr. Biol..

[20] Wiecki, T. V., Sofer, I. & Frank, M. J. HDDM: Hierarchical Bayesian estimation of the Drift-Diffusion Model in Python. *Front. Neuroinform.* **7** (2013). 10.3389/fninf.2013.00014 PMC3731670 23935581

[21] Tusche A, Hutcherson CA (2018). Cognitive regulation alters social and dietary choice by changing attribute representations in domain-general and domain-specific brain circuits. Elife.

[22] Ratcliff R, Tuerlinckx F (2002). Estimating parameters of the diffusion model: Approaches to dealing with contaminant reaction times and parameter variability. Psychon. Bull. Rev..

[23] Spiegelhalter DJ, Best NG, Carlin BP, Linde AVD (2002). Bayesian measures of model complexity and fit. J. R. Stat. Soc. Ser. B (Stat. Methodol.).

[24] Palminteri S, Wyart V, Koechlin E (2017). The importance of falsification in computational cognitive modeling. Trends Cognit. Sci..

[25] Gelman A, Rubin DB (1992). Inference from iterative simulation using multiple sequences. Stat. Sci..

[26] Kruschke JK (2013). Bayesian estimation supersedes the t test. J. Exp. Psychol. Gen..

[27] Lobel TE (2015). Being clean and acting dirty: The paradoxical effect of self-cleansing. Ethics Behav..

[28] Yu H, Siegel JZ, Crockett MJ (2019). Modeling morality in 3-D: decision-making, judgment, and inference. Top. Cogn. Sci..

[29] Hutcherson CA, Montaser-Kouhsari L, Woodward J, Rangel A (2015). Emotional and utilitarian appraisals of moral dilemmas are encoded in separate areas and integrated in ventromedial prefrontal cortex. J. Neurosci..

[30] Shenhav A, Greene JD (2010). Moral judgments recruit domain-general valuation mechanisms to integrate representations of probability and magnitude. Neuron.

[31] Andreoni J (1990). Impure altruism and donations to public goods: A theory of warm-glow giving. Econ. J..

[32] Dana J, Weber RA, Kuang JX (2007). Exploiting moral wiggle room: experiments demonstrating an illusory preference for fairness. Econ. Theor..

[33] Volz LJ, Welborn BL, Gobel MS, Gazzaniga MS, Grafton ST (2017). Harm to self outweighs benefit to others in moral decision making. PNAS.

[34] van der Plas E, David AS, Fleming SM (2019). Advice-taking as a bridge between decision neuroscience and mental capacity. Int. J. Law Psychiatry.

[35] Berman JZ, Kupor D (2020). Moral choice when harming is unavoidable. Psychol Sci.

[36] van Vugt, M., Simen, P., Nystrom, L., Holmes, P. & Cohen, J. EEG Oscillations Reveal Neural Correlates of Evidence Accumulation. *Frontiers Neurosci.* **6** (2012). 10.3389/fnins.2012.00106 PMC3398314 22822389

[37] Lockwood PL (2017). Prosocial apathy for helping others when effort is required. Nat Hum Behav.

[38] Hare TA, Malmaud J, Rangel A (2011). Focusing attention on the health aspects of foods changes value signals in vmPFC and improves dietary choice. J. Neurosci..

[39] Smith SM, Krajbich I (2018). Attention and choice across domains. J. Exp. Psychol. Gen..

[40] Bénabou, R., Falk, A. & Tirole, J. *Narratives, Imperatives, and Moral Reasoning*. https://www.nber.org/papers/w24798 (2018). 10.3386/w24798.

