# Supplementary material for: A computational account of how individuals resolve the dilemma of dirty money
Source: Sci Rep. 2022 Nov 3;12:18638. doi: 10.1038/s41598-022-22226-9 (PMC9633827; doi:10.1038/s41598-022-22226-9)
Supplement: Supplementary file 1 — Supplementary Information. [file 41598_2022_22226_MOESM1_ESM.pdf]

Resolving the dilemma of dirty money: a computational account
Supplementary Material

Model Parameter Details

Our Drift Diffusion Model (DDM) jointly considers choices and response time (RT) to infer latent cognitive parameters. The DDM captures the efficiency at which information about the choice attributes (i.e. shocks and money) are processed with the drift rate parameter, *v*. Once the drift rate crosses a threshold, the corresponding option (larger or smaller shock and money offers) is chosen. Previous research has consistently linked the drift rate parameter with attentional processes [S1–4]. More specifically, eye-tracking data (gaze, fixations) often track the direction of evidence accumulation. Furthermore, individuals with clinical symptoms of inattention (attention deficit hyperactivity disorder) appear to have lower drift rates in non-social binary decision making tasks [S2,5]. Other work has shown that manipulating which features of a decision people attend to impacts drift rates in the DDM [S6]. Cumulatively, this work suggests that shifts in drift rate reflect shifts in attentional processes involved in complex decision-making.

Because information about money makes people generally more willing to accept larger shock and money offers, whilst information about shocks makes people generally less willing to accept larger shock and money offers*,* money and shocks push *v*, the accumulation, in opposite directions. Individual differences in how much weight someone places on either choice attribute affects how likely they are to choose either option. For example, the accumulation process of a person with larger weights on shocks is more likely to result in faster and more choices for the lower shocks and money offer.

The bias parameter, *z*, reflects an a-priori bias in the decision process towards a specific action. For instance, when asked to decide whether a stimulus is threatening or safe, individuals with anxiety show a bias towards responding that a stimulus is threatening, regardless of what stimulus is shown [S7]. In turn, the bias parameter within the current paradigm captures the extent to which people lean toward accepting the larger amount of money before they know how much money is at stake. Importantly, as the bias parameter is distinct from the drift rate (evidence accumulation) parameter, it indicates an a priori preference for the larger or smaller shock and money offer. A consequence of an a priori bias towards the lower shocks and money offer are, on average, faster and more frequent choices for the lower shocks and money offer.

Given that several latent parameters influence choice behavior, the same choice frequency can arise from either a change in the drift rate or a change in the starting point. Figure S1 (modified from [S8] schematically illustrates how the DDM has multiple latent parameters to account for changes in observed choice probabilities. The top row shows a baseline set of parameter values (left), an increase in starting point towards acceptance of more money and more shocks (middle), and a change in drift rate towards acceptance of more money and more shocks (right). These two parameter changes lead to the same change in choice behavior across trials (bottom left), but create clear differences in the distribution of RT (bottom right). A clear change in the positive skew of the RT distribution for a change in starting point is observed relative to the RT distribution for a change in drift rate. Thus, looking at both choice and RT allows the DDM to better identify the process driving the change in choice behavior.

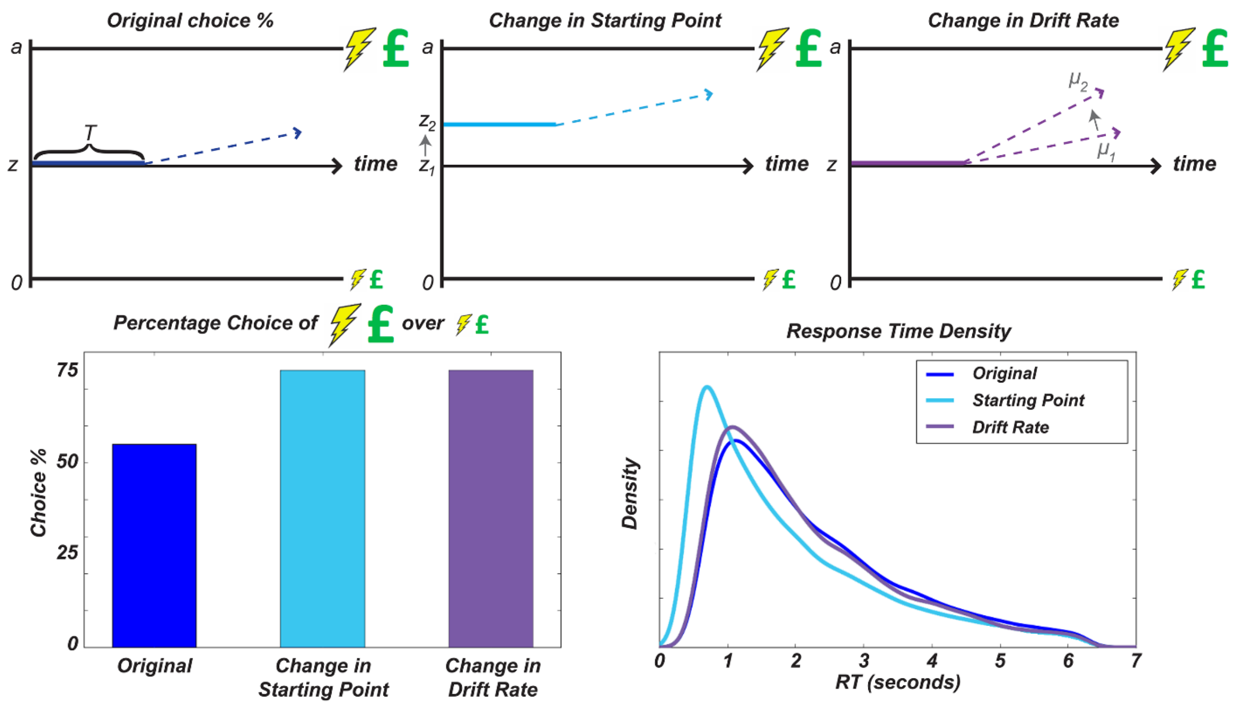

Figure S1. *Schematic showing how multiple latent parameters can explain changes in observed choice probabilities. Figured and caption modified from* [8].

Model Comparison Details

To address which HDDM specification could best account for behavior arising from source effects (Studies 1 and 3) and destination effects (Study 3), we used two metrics for model comparison. As discussed in the main text, we used the Deviance Information Criterion (DIC) and mean-squared error (MSE) between observed data and calibrated simulations. As a baseline, we fit a naïve model for which parameters did not vary at all by condition or trial (Base). The *prosocial default* (PD) model fit separate starting points z for trials depending on whether or not the money was dirty or clean (i.e., source effects). For the *valuation conflict* (VC) hypothesis, we fit four models, using the same logic for both Study 1 and Study 3, under the assumption that Δm and Δs are integrated during the accumulation process, and thus captured through a weighted drift rate v.

For Study 1, the models are as follows:

- V1, which has a single regressor for money and a single regressor for shocks, not differentiating on their source: β_m, β_s
- V2, which has two regressors for money and a single regressor for shocks, assuming source effects only apply to money: $\beta_{m,clean}, \beta_{m,dirty}, \beta_s$
- V3, which has one regressor for money and two regressors for shocks, assuming source effects only apply to shocks: $\beta_m, \beta_{s,clean}, \beta_{s,dirty}$
- V4, which has two regressors for money and two regressors for shocks, assuming source effects apply to both money and shocks: $\beta_{m,clean}, \beta_{m,dirty}, \beta_{s,clean}, \beta_{s,dirty}$

As can be seen in Table S1, V4 was the best-performing model in all three categories for Study 1: DIC, choice data MSE, and RT data MSE. So, it is the valuation conflict model referenced in the main text for Study 1.

For Study 3, the models are as follows. The two "intermediate" models, V2 and V3, build on the results of Study 1 and assume only source effects on the Profit condition.

- V1, which has a single regressor for money and a single regressor for shocks, not differentiating on their source: β_m, β_s
- V2, which has three regressors for money. Destination effects for all money, but source effects only when the money is for Profit. The model has a single regressor for shocks, assuming neither source nor destination effects apply to shocks.
$$\beta_{m,clean\ profit}, \beta_{m,dirty\ profit}, \beta_{m,charity}, \beta_s$$
- V3, which has one regressor for money and three regressors for shocks, assuming neither source nor destination effects apply to money. Destination effects for all shocks, but source effects on shocks only when the money is for Profit.
$$\beta_m, \beta_{s,clean\ profit}, \beta_{s,dirty\ profit}, \beta_{s,charity}$$
- V4, which has four regressors for money and fours regressors for shocks, assuming source and destination effects apply to both money and shocks:
$$\beta_{m,clean\ profit}, \beta_{m,clean\ charity}, \beta_{m,dirty\ profit}, \beta_{m,dirty\ chartiy}$$
$$\beta_{s,clean\ profit}, \beta_{s,clean\ charity}, \beta_{s,dirty\ profit}, \beta_{s,dirty\ chartiy}$$

As can be seen in Table S1, V4 was the best-performing model in all three categories for Study 3: DIC, choice data MSE, and RT data MSE. So, it is the valuation conflict model referenced in the main text for Study 3.

**Study 1**				**Study 3**			
**Model**	**DIC**	**MSE Choice**	**MSE RT**	**Model**	**DIC**	**MSE Choice**	**MSE RT**
Base	20648.637	0.2171	0.7702	Base	35520.081	0.2666	0.6413
PD	20635.438	0.2163	0.7695	MC	35535.331	0.2499	0.6355
VC1	16724.755	0.1003	0.6831	VC1	28243.571	0.1202	0.5944
VC2	16656.746	0.1015	0.6896	VC2	28057.741	0.1177	0.5904
VC3	16674.947	0.1020	0.6860	VC3	28089.82	0.1177	0.5915
VC4	16653.221	0.0992	0.6788	VC4	28023.198	0.1170	0.5900
VC4+PD	16662.657	0.0993	0.6792	VC4+MC	28091.39	0.1171	0.5910

Table S1. Summary of HDDM model comparison for Study 1 (left columns) and Study 3 (right columns).

Model Parameter Summaries

For both Study 1 and Study 3, the best performing model corresponded to the valuation conflict model (VC4 in **Table S1**). **Table S2** and **Table S3** summarize the group-level parameter estimates for those models

**Parameter**	**Mean**	**SD**
Drift (v), Intercept	0.154	0.101
Drift (v), Money Clean	0.097	0.002
Drift (v), Money Dirty	0.084	0.002
Drift (v), Shock Clean	-0.09	0.002
Drift (v), Shock Dirty	-0.096	0.003
Barrier (a)	3.082	0.103
Starting Point (z)	0.504	0.008
Non-Decision Time (t)	0.539	0.051
**Convergence**	**Mean**	**SD**
Rhat	1.0005	0.0007

Table S2. DDM parameter estimates for Study 1. Statistics reflect the mean and standard deviation (SD) of the N=10,000 posterior samples of group-level parameters. Model convergence is summarized with the mean and standard deviation (SD) of $\hat{R}$ for all model parameters.

Parameter	Mean	SD
Drift (v), Intercept	0.279	0.087
Drift (v), Money Clean Profit	0.090	0.003
Drift (v), Money Clean Charity	0.067	0.003
Drift (v), Money Dirty Profit	0.066	0.003
Drift (v), Money Dirty Charity	0.068	0.002
Drift (v), Shock Clean Profit	-0.052	0.002
Drift (v), Shock Clean Charity	-0.070	0.002
Drift (v), Shock Dirty Profit	-0.055	0.002
Drift (v), Shock Dirty Charity	-0.064	0.002
Barrier (a)	2.752	0.057
Starting Point (z)	0.506	0.010
Non-Decision Time (t)	0.384	0.018
**Convergence**	**Mean**	**SD**
Rhat	1.0002	0.0003

Table S3. DDM parameter estimates for Study 3. Statistics reflect the mean and standard deviation (SD) of the N =10,000 posterior samples of group-level parameters. Model convergence is summarized with the mean and standard deviation (SD) of $\hat{R}$ for all model parameters.

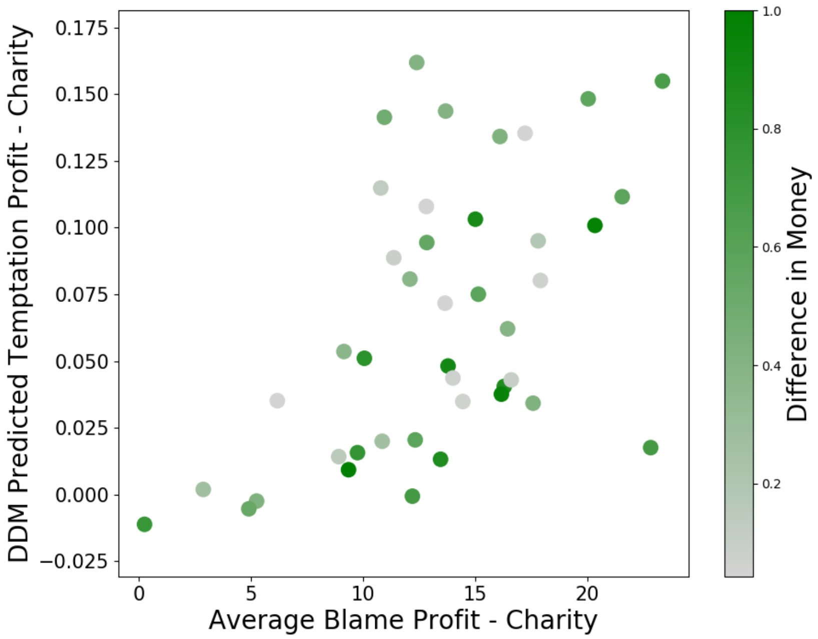

Figure S2. DDM parameter. This figure corresponds to **Figure 4d** in the main text. The only difference is the color coding of each dot by the difference in money (Δm) for the two options.

Regression Results for Blame Judgments in Study 2

	estimate	s.e.m.	t-stat	p-value	lower CI	upper CI
Intercept	64.752	1.948	33.248	<0.000	60.934	68.570
Money	-1.879	0.078	-24.211	<0.000	-2.031	-1.727
Harm	1.697	0.081	20.894	<0.000	1.538	1.856
Condition	-8.237	2.733	-3.014	<0.003	-13.595	-2.879
Money X Condition	-0.090	0.109	-0.830	0.406	-0.304	0.123
Harm X Condition	-0.503	0.114	-4.415	<0.000	-0.727	-0.280

Table S4. Complete results from the regression analysis on blame judgements in Study 2. we used a linear mixed effects model with a random intercept to model how money and shocks in each choice option predict blame judgements, and whether the effects of money and shocks on blame judgments differ as a function of the money recipient.

References

S1. Cavanagh, J. F., Wiecki, T. V., Kochar, A. & Frank, M. J. Eye tracking and pupillometry are indicators of dissociable latent decision processes. *Journal of Experimental Psychology: General* **143**, 1476–1488 (2014).

S2. Feldman, J. S. & Huang-Pollock, C. Slow drift rate predicts ADHD symptomology over and above executive dysfunction. *Child Neuropsychol* **27**, 834–855 (2021).

S3. Haller, S. P. *et al.* Computational Modeling of Attentional Impairments in Disruptive Mood Dysregulation and Attention-Deficit/Hyperactivity Disorder. *Journal of the American Academy of Child & Adolescent Psychiatry* **60**, 637–645 (2021).

S4. Smith, S. M. & Krajbich, I. Gaze Amplifies Value in Decision Making. *Psychol Sci* **30**, 116–128 (2019).

S5. Metin, B. *et al.* ADHD performance reflects inefficient but not impulsive information processing: A diffusion model analysis. *Neuropsychology* **27**, 193–200 (2013).

S6. Smith & Krajbich. Attention and choice across domains. *Journal of Experimental Psychology: General* **147**, 1810 (2018).

S7. White, C. N., Skokin, K., Carlos, B. & Weaver, A. Using decision models to decompose anxiety-related bias in threat classification. *Emotion* **16**, 196–207 (2016).

S8. Clithero, J. A. Improving out-of-sample predictions using response times and a model of the decision process. *Journal of Economic Behavior & Organization* **148**, 344–375 (2018).
